# Quantifying the importance of disease burden on perceived general health and depressive symptoms in patients within the Mayo Clinic Biobank

**DOI:** 10.1186/s12955-015-0285-6

**Published:** 2015-07-03

**Authors:** Euijung Ryu, Paul Y. Takahashi, Janet E. Olson, Matthew A. Hathcock, Paul J. Novotny, Jyotishman Pathak, Suzette J. Bielinski, James R. Cerhan, Jeff A. Sloan

**Affiliations:** Department of Health Sciences Research, Rochester, MN USA; Department of Medicine, Mayo Clinic, Rochester, MN USA

**Keywords:** Health-related quality of life, Biobank, Disease burden, Relative influence

## Abstract

**Background:**

Deficits in health-related quality of life (HRQOL) may be associated with worse patient experiences, outcomes and even survival. While there exists evidence to identify risk factors associated with deficits in HRQOL among patients with individual medical conditions such as cancer, it is less well established in more general populations without attention to specific illnesses. This study used patients with a wide range of medical conditions to identify contributors with the greatest influence on HRQOL deficits.

**Methods:**

Self-perceived general health and depressive symptoms were assessed using data from 21,736 Mayo Clinic Biobank (MCB) participants. Each domain was dichotomized into categories related to poor health: deficit (poor/fair for general health and ≥3 for PHQ-2 depressive symptoms) or non-deficit. Logistic regression models were used to test the association of commonly collected demographic characteristics and disease burden with each HRQOL domain, adjusting for age and gender. Gradient boosting machine (GBM) models were applied to quantify the relative influence of contributors on each HRQOL domain.

**Results:**

The prevalence of participants with a deficit was 9.5 % for perception of general health and 4.6 % for depressive symptoms. For both groups, disease burden had the strongest influence for deficit in HRQOL (63 % for general health and 42 % for depressive symptoms). For depressive symptoms, age was equally influential. The prevalence of a deficit in general health increased slightly with age for males, but remained stable across age for females. Deficit in depressive symptoms was inversely associated with age. For both HRQOL domains, risk of a deficit was associated with higher disease burden, lower levels of education, no alcohol consumption, smoking, and obesity. Subjects with deficits were less likely to report that they were currently working for pay than those without a deficit; this association was stronger among males than females.

**Conclusions:**

Comorbid health burden has the strongest influence on deficits in self-perceived general health, while demographic factors show relatively minimal impact. For depressive symptoms, both age and comorbid health burden were equally important, with decreasing deficits in depressive symptoms with increasing age. For interpreting patient-reported metrics and comparison, one must account for comorbid health burden.

## Introduction

Health-related quality of life (HRQOL) describes a patient’s perception of how his or her health status affects physical, psychological, and social functioning and well-being [1, 2]. The focus for health care has become increasingly aimed at HRQOL as well as the quantity of life bestowed by clinical treatments [3]. This is especially true among chronic disease populations where cure remains elusive. In particular, cancer care frequently measures HRQOL as an important outcome [4, 5]. Even for diseases where cures are more routine, the impact on HRQOL of the patient has been shown to be related to treatment outcome [4, 6, 7]. Recent studies showed that HRQOL can be prognostic of survival and other treatment outcomes as well as useful in identification of otherwise undetected clinical problems [4, 7].

Along with many HRQOL items, perceived general health and depressive symptoms have received increased attention for potential clinical relevance. Perceived general health (i.e., self-assessed health) has been recognized as a valuable clinical tool, as it captures both current health status and subtle changes in health [8]. Recent studies reported that overall perception of general health can be used as a quick tool for identifying patients at high risk of imminent death and hospitalization [9]. Depressive symptoms are also known to have a potentially large influence on overall clinical outcomes including survival [10]. Patients with depressive symptoms are less likely to seek treatment for medical conditions and to adhere to treatment recommendations [11–13], and thus have potentially worse clinical outcomes.

There are gender differences in both the frequency and manner in which patients report clinical symptoms and treatment-related side effects [4]. These reporting differences may affect self-reported HRQOL. Men have been observed to communicate their needs less than female counterparts and risk failing to acknowledge existing medical problems until the window of opportunity for effective intervention has expired [14, 15]. It is less studied whether predictors of HRQOL are the same among men and women, and how the impacts of these predictors are influenced by gender.

Most research on HRQOL predictors focus on patients with individual diseases. Some predictors include socioeconomic status, age, gender, and comorbid health burden. A study based on pediatric patients with diabetes showed that HRQOL is influenced not only by disease-related factors but by the complex of non-disease related determinants such as gender and socioeconomic status [16]. In terms of determinants not directly related to a specific disease symptom, arthritis-related pain is most prevalent among patients aged between 45 and 64, blacks and Hispanics, and with less than a high school education [17]. A study using patients with Type 1 diabetes and coeliac disease showed an impact of multiple comorbidities on HRQOL [18]. Those with both conditions had significantly lower HRQOL than patients with only Type 1 diabetes alone. The impact of multiple comorbidities on HRQOL is well supported by numerous studies [19–22]. For instance, a recent study based on Medicare beneficiaries showed that the majority of chronic conditions, including cancer, were associated with decrements in HRQO, with substantial impact of the cumulative effects of comorbid conditions [19]. However, in broad patient populations without specific illnesses, the predictors of HRQOL are relatively less known. Furthermore, it is not yet quantified the relative influence of HRQOL of each determinant such as comorbid health burden, age, gender, lifestyle issues, and body mass index (BMI) in the general population.

Using patients with a wide range of medical conditions, this study was to evaluate the impact of commonly collected health and lifestyle determinants for HRQOL deficits. More importantly, we aimed to quantify the relative influence of each determinant when all of these determinants are simultaneously considered. As a secondary aim, we assessed gender as a potential modifier of the contributors.

## Materials and methods

### Participants and setting

This study was reviewed and approved by the Mayo Clinic Institutional Review Board (IRB). All participants from the study were from the Mayo Clinic Biobank (MCB). The MCB is an institutional resource initiated by the Mayo Clinic Center for Individualized Medicine [23–25]. Enrollment into the MCB began in April 2009 and is ongoing with a target goal of 50,000 participants. Eligible subjects were 18 years of age or older, able to provide informed consent, had ever been a patient at Mayo Clinic and were residents of the US. Of those, patients with a medical appointment mainly in internal and family medicine departments were invited to the MCB via mailed invitation, while allowing volunteers. Among those invited, 29 % participated, 15 % refused, and the remainder did not respond to the invitations. More details regarding enrollment will be found in the MCB design paper [23].

Unlike disease-specific biobanks, the MCB selects participants based neither on disease nor on exposure history, but rather on some other selection factors such as location of residence or source of clinical care [23]. At enrollment into the MCB, the participants provided consent to utilize biological samples and to access to their electronic medical records (EMR) for research studies approved by IRB and the MCB access committee, and completed self-reported health information questionnaires including HRQOL-related domains [23]. A copy of the current version of questionnaires is available at the MCB website (http://www.mayo.edu/research/documents/biobank-questionnaire/doc-20086430). In the current study, we used the data from 21,736 MCB participants recruited in the first 3 years of the enrollment (April 2009 through March 2012).

### HRQOL-related outcomes

We considered two HRQOL-related domains included in the MCB baseline questionnaire. Perception of general health was obtained by a single question, “In general, would you say your health is excellent, very good, good, fair or poor.” Poor or fair perceived health was considered as a deficit. Depressive symptoms were assessed using self-reported frequency of depressed mood and anhedonia using two PHQ-2 questions (“During the past 2 weeks, how often have you been bothered by feeling down, depressed, or hopeless?” and “During the past 2 weeks, how often have you been bothered by having little interest or little pleasure in doing things?”), scoring each as 0 (“not at all”) to 3 (“nearly every day”) [26]. Combined scores with at least 3 were considered as a deficit. In addition to these two HRQOL-related domains, overall quality of life (QOL) rating was also obtained by a single question, “How would you describe your overall quality of life?”, rating 0 (“as bad as it can be”) to 10 (“as good as it can be”).

### Predictors

#### Demographic characteristics

Age at the MCB enrollment and gender were obtained from the institutional patient registration database. In the baseline questionnaire completed at enrollment, participants self-reported level of education (high school graduate or less, some college, Bachelor’s degree, or graduate school degree), employment status (currently working, retired, or not working for other reasons), alcohol consumption (at least 2 or more times per week, once a week or less, never or less than once a month), and tobacco smoking (at least 100 cigarettes in lifetime, yes/no). To calculate BMI, height (in meters) and weight (in kilograms) were extracted from the EMR. Measurements closest, but prior, to the enrollment were selected if available. If no EMR data were available within 2 years prior to enrollment, self-reported height and weight data were used to calculate BMI (kg/m^2^). BMI was categorized into 4 groups (underweight: <18.5, normal: 18.5 – 24.9, overweight: 25 – 29.9, obese: 30+).

#### Self-reported diseases and disease burden

Also included in the baseline questionnaire was a series of questions on comorbidities at the time of enrollment. We obtained the presence of illness and age at diagnosis with 80 diseases from 11 different disease categories (rheumatologic, liver, hematologic, cancer, neurologic, mental health, eye, cardiovascular, respiratory, gastrointestinal, and endocrine). Non-melanoma skin cancer was separated from other cancers. Disease burden was measured by the total number of reported diseases, which has been shown to be positively associated with healthcare cost [27].

### Statistical analyses

To compare subjects with and without HRQOL deficits, we performed Pearson chi-square tests to assess the association of each categorical characteristic. Mann–Whitney tests were used for continuous characteristics (age and disease burden). To examine potential gender differences in deficits, the gender-specific proportion of subjects with deficits in each age group (<45, 45 – 54, 55–64, 65+) was calculated for both perceived general health and depressive symptoms. Correlation between perceived general health and depressive symptoms were calculated using Cohen’s kappa statistics. In addition, associations between these two HRQOL-related domains and the overall QOL were tested using Mann–Whitney *U*-test.

Adjusting for age by using natural cubic splines, we applied logistic regression models to test the association of each HRQOL measure with demographic characteristics listed in Table 1 and disease burden, stratified by gender. Due to potential correlation between disease burden and BMI and/or employment status, the analysis was repeated after adjusting for disease burden. We tested whether gender was a modifier by including an interaction term between gender and each predictor in logistic regression models. To further investigate the impact of disease burden, the association between a given disease group and risk of HRQOL deficits was tested using logistic regression models, adjusting for age and gender. Odds ratio (OR) and its 95 % confidence interval (CI) were presented for the association of each variable.

**Table 1:** Characteristics of the Mayo Clinic Biobank participants, for all and two HRQOL-related domains

	Overall cohort (*n* = 21,736)	Perceived general health			Depressive symptoms		
		Deficits (*n* = 2079)	Non-deficits (*n* = 19,657)	*P*-value*	Deficits (*n* = 1009)	Non-deficits (*n* = 20,727)	*P*-value**
Age,				<0.001			<0.001
Median (25^th^ – 75^th^ % tiles)	62 (52,72)	63 (52,74)	62 (52,74)		55 (45,66)	63 (52,72)	
Female %	57 %	58 %	57 %	0.52	61 %	57 %	0.03
Education				<0.001			<0.001
High school or less	18 %	28 %	17 %		24 %	18 %	
Some college	33 %	40 %	32 %		40 %	33 %	
Bachelor degree	25 %	16 %	26 %		17 %	25 %	
Graduate school	24 %	16 %	25 %		19 %	24 %	
Employment				<0.001			<0.001
Not working - retired	34 %	41 %	33 %		25 %	34 %	
Not working - others	12 %	29 %	10 %		30 %	11 %	
Currently working	54 %	30 %	56 %		44 %	54 %	
BMI				<0.001			<0.001
Underweight	2.0 %	3.6 %	1.8 %		3.3 %	2.0 %	
Normal	28 %	24 %	29 %		25 %	28 %	
Overweight	37 %	26 %	37 %		28 %	37 %	
Obese	33 %	47 %	32 %		43 %	33 %	
Smoking, ever %	42 %	54 %	41 %	<0.001	54 %	42 %	<0.001
Alcohol				<0.001			<0.001
Never or less than once a month	42 %	64 %	39 %		57 %	41 %	
Once a week or less	23 %	17 %	24 %		19 %	23 %	
2+ a week	35 %	19 %	37 %		24 %	36 %	
Disease burden (Number of self-reported diseases),				<0.001			<0.001
Median (25^th^ – 75^th^ % tiles)	4 (2,6)	8 (5,10)	4 (2,6)		6 (4,9)	4 (2,6)	

*, **P-values comparing subjects with and without deficits in perceived general health (*), and depressive symptoms (**)

Among all predictors considered, relative influence of each variable on risk of deficits in each HRQOL was estimated by applying gradient boosting machine (GBM) models, allowing interaction by gender [28, 29]. To evaluate prediction accuracy of the GBM models, 10-fold cross-validation approaches were used and average C-statistics and average relative influences of the most informative variable were presented. The GBM modeling approach is a machine learning technique for building a multivariable prediction model by incorporating all of the variables without variable selection. This approach has been reported to be least affected by overfitting compared to other popular machine learning methods such as neural networks and support vector machines [28, 30]. In addition, the GBM modelling has the advantage over regression models because it can easily capture non-linearity of continuous variables and interaction terms among the variables without prior specification [28].

## Results

### Participants

Among 21,736 participants, median age at enrollment was 62 years and 57 % were female. We found 49 % had at least a Bachelor’s degree and 54 % were currently employed. Other demographic characteristics of the overall cohort are noted in Table 1. Median of disease burden (the number of self-reported diseases) was four (Table 1). The proportion of participants with deficits was 9.5 % (n = 2079) for perception of general health and 4.6 % (n = 1009) for depressive symptoms. Slight agreement was observed between the two HRQOL measures (Cohen’s kappa = 0.23). These two measures were strongly associated with the overall QOL ratings, with worse overall QOL among those with deficits in HRQOL measures (median overall QOL = 8 for subjects with deficits, compared to 5 for those with non-deficits in each domain; p-values < 0.001).

### Age and HRQOL

**Fig. 1:**
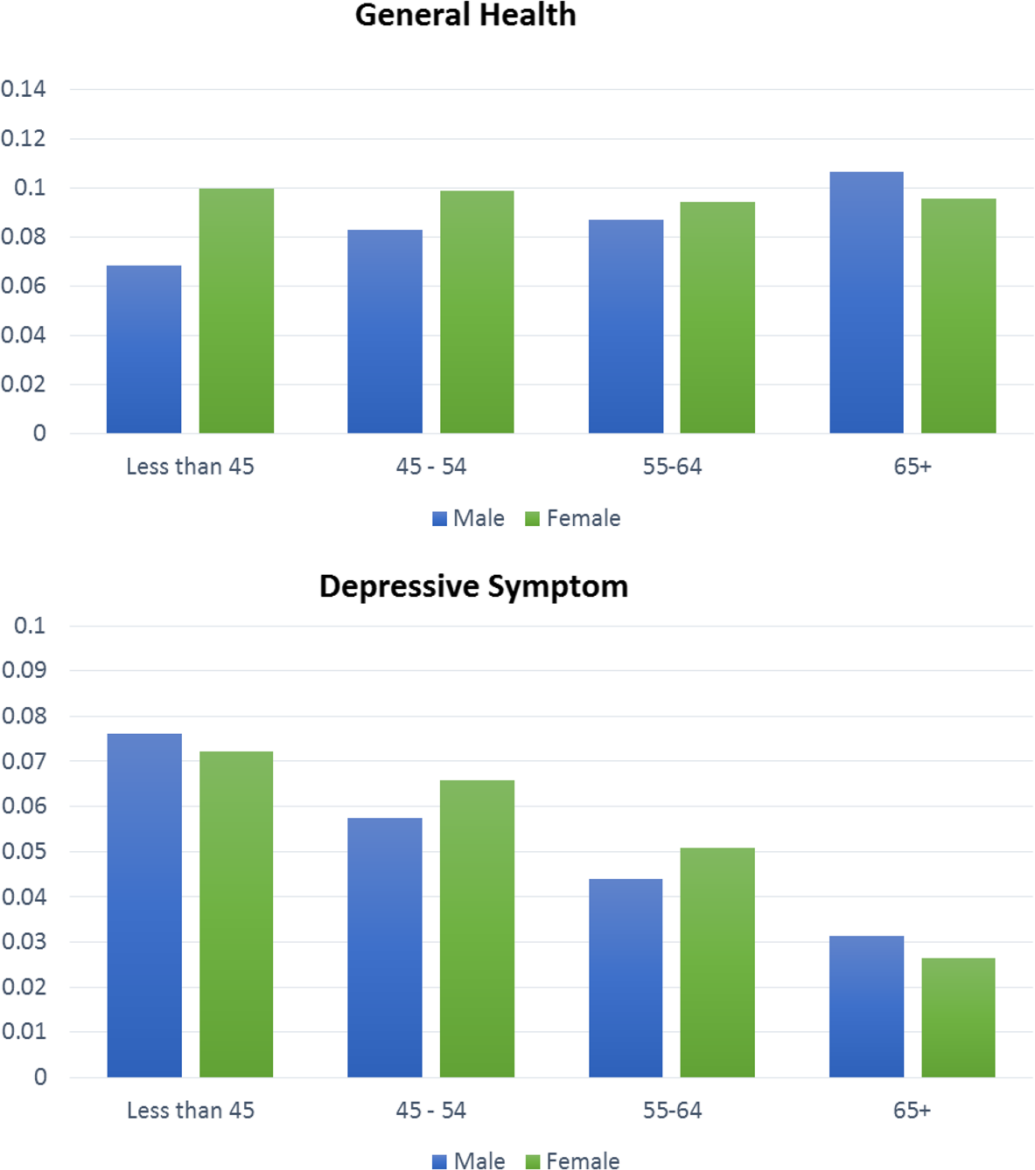
Relationship between age and the proportion of deficits in two HRQOL-related domains, stratified by gender

Subjects with deficits in perceived general health were slightly older than those without deficits (median age of 63 vs 62 years). There was an inverse association with age and the prevalence of deficits in depression symptoms (median age of 55 in those with deficits vs 63 years in those without). For perceived general health, the prevalence of deficits slightly increased with age for males (6.8 % vs 11 % for subjects aged ≤ 45 years vs. 65 years or older), while the prevalence was similar across age groups for females (10 % for all ages). The proportion of deficits in depressive symptoms decreased with age for both genders (Fig. 1).

### Disease burden and HRQOL

**Fig. 2:**
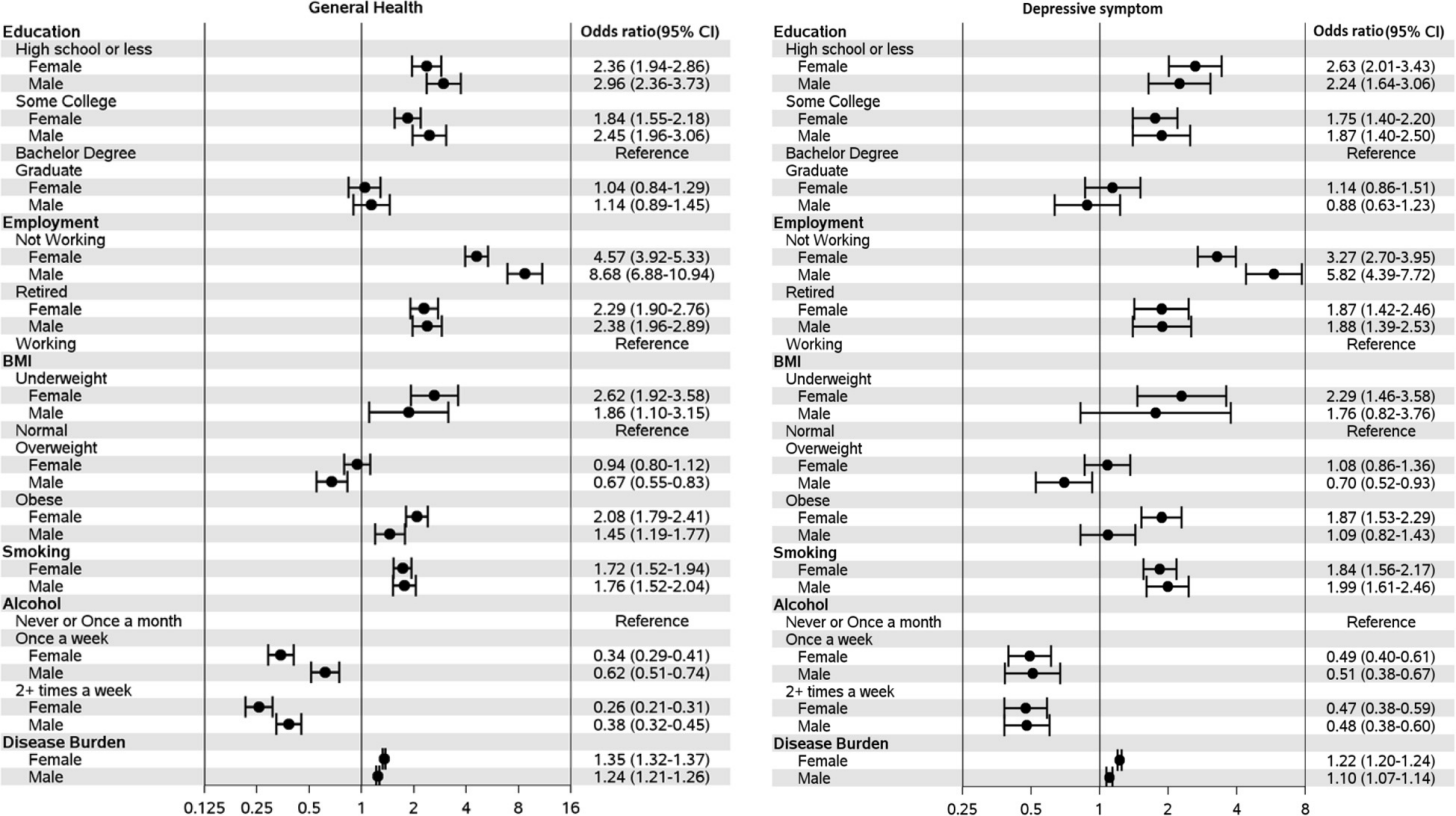
Association of participant characteristics with risk of deficits in two HRQOL-related domains, stratified by gender. Age-adjusted odds ratio and 95 % confidence intervals (95 % CIs) are presented for each characteristic

**Fig. 3:**
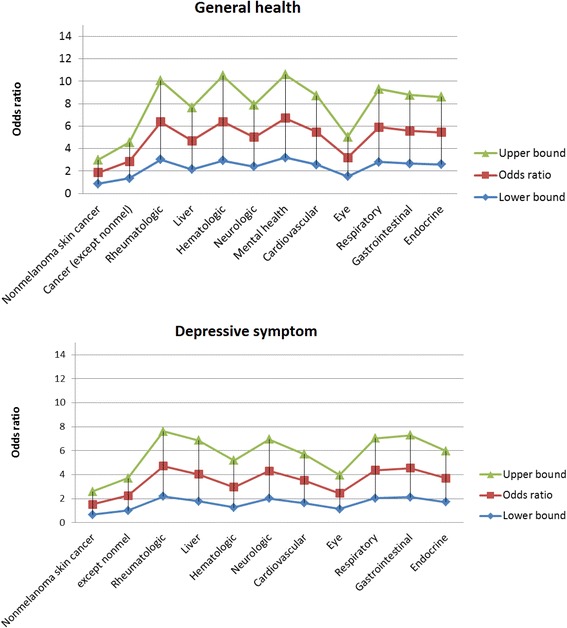
Association of each disease category with risk of deficits in two HRQOL-related domains. Age-gender-adjusted odds ratio and 95 % CIs (upper bounds in green and lower bound in blue) are presented for each disease category. For depressive symptoms, association with mental health condition was excluded

Disease burden was strongly associated with higher risk of HRQOL deficits, with a stronger association seen with the risk of deficits in perceived general health (OR = 1.3, 95 % CI 1.29 – 1.32 for perceived general health; OR = 1.2, 95 % CI 1.16 – 1.20 for deficits in depressive symptoms). The association of disease burden with risk of deficits was stronger among females compared to males for both HRQOL measures (Fig. 2). Among 12 disease groups used to calculate disease burden, cardiovascular diseases (which includes hypertension and hyperlipidemia) were most prevalent among the MCB participants (67 % in males, 53 % in females). Diseases that were more prevalent in females included rheumatologic (41 % vs 31 %), neurologic (28 % vs 16 %), mental health (33 % vs 21 %), and endocrine disorders (32 % vs 17 %). Except for non-melanoma skin cancer, all disease groups showed a strong association with risk of deficits in both HRQOL measures (Fig. 3).

### Demographic characteristics and HRQOL

For both general health and depressive symptom, risks for deficits were associated with lower level of education, ever smoking, and no alcohol consumption (Table 1, Fig. 2). Subjects with deficits in perceived general health were less likely to report that they were currently working for pay (OR = 5.7, 95 % CI 5.1 – 6.5, when comparing subjects not currently working for pay vs those working for pay), with a stronger association among males (Fig. 2, P-value for gender interaction < 0.001). A similar pattern of association was observed for deficits in depressive symptoms (OR = 4.0, 95 % CI 3.4 – 4.7 overall, with stronger association among males, Fig. 2). We also examined whether adjusting for disease burden would alter the strength of the association for employment status and HRQOL deficits. Although the associations were attenuated, they remained statistically significant for the risk of deficits in both HRQOL measures (for the perceived general health, OR = 5.8 [95 % CI 4.5 – 7.5] for males and 2.9 [95 % CI 2.4 – 3.4] for females; for depressive symptoms, OR = 3.7 [2.7 – 5.0] for males and 1.9 [95 % CI 1.5 – 2.3] for females).

### Body mass index and HRQOL

Compared to subjects with normal weight, obesity was positively associated with higher risk of deficits in perceived general health (OR = 1.9, 95 % CI 1.7 -2.1), especially among females (OR = 2.1, 95 % CI 1.8 – 2.4 for females; OR = 1.5, 95 % CI 1.2 – 1.8 for males, P-value for gender interaction = 0.005). Obesity was positively associated with deficits in depressive symptoms among females (OR = 1.9, 95 % CI 1.5 – 2.3), but not in males (OR = 0.7, 95 % CI 0.8 – 1.4; P-value for gender interaction = 0.01). Overweight males tended to have lower risk of HRQOL deficits than normal weight males in both HRQOL-related domains, while the risk of deficits in both domains were similar between normal and overweight females (Fig. 2). Once adjusted for disease burden, the association of obesity with risk of HRQOL deficits noted above was no longer observed. However, the protective effect of overweight among males still remained even after adjusting for disease burden (OR = 0.6, 95 % CI 0.5 - 0.7 for deficits in perceived general health; OR = 0.6, 95 % CI 0.6 – 0.9 for deficits in depressive symptoms).

### Relative influence of each predictor on HRQOL

**Fig. 4:**
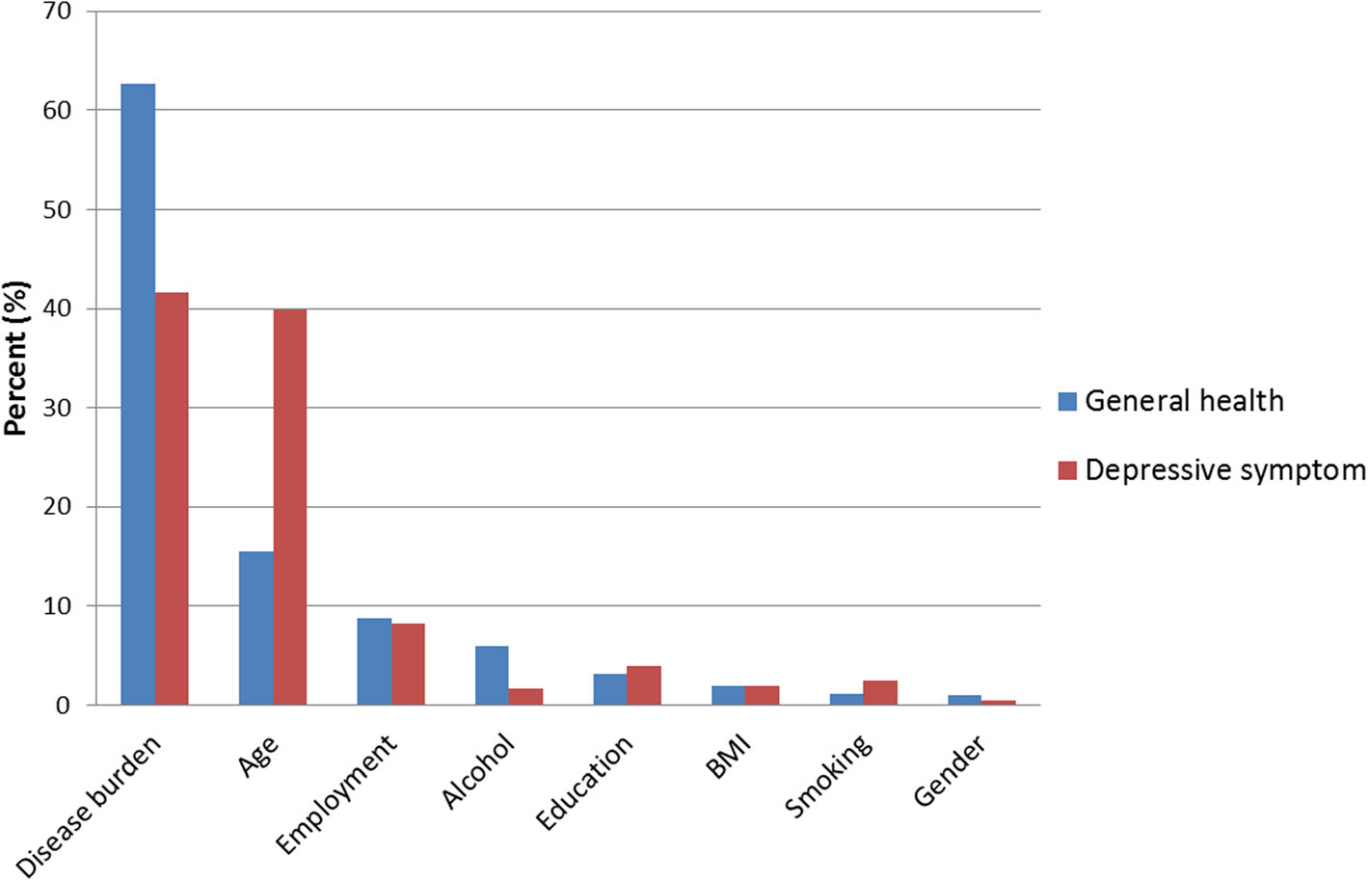
Relative influence (%) of various characteristics for risk of deficits in two HRQOL-related domains

Figure 4 shows the relative influence of various factors on the risk of deficits in perceived general health and depressive symptoms. Among all the predictors considered, disease burden had the greatest influence (63 %) on risk of deficits in perceived general health, followed distantly by age (16 %). For depressive symptoms, disease burden and age showed similar influence on risk of deficits (42 % and 40 %, respectively). The average relative influence of disease burden from 10-fold cross-validation was xx (ranging from xx to xx) for perceived general health, and 42 % (ranging from 41 % to 43 %) for depressive symptoms. The average C-statistics was 0.82 and 0.77 for perceived general health and depressive symptoms, respectively. For both HRQOL-related domains, influences of demographic factors were minimal (<10 %) in presence of disease burden, although each had strong association with HRQOL deficits individually.

## Discussion

Using a broad patient population enrolled into the Mayo Clinic Biobank, we found that prevalence of deficits in perceived general health (10 % with poor or fair health) and depressive symptoms (5 % with PHQ-2 score 3 or higher) was relatively low. The Center for Disease Control’s 2010 Behavioral Risk Factor Surveillance System Survey (http://apps.nccd.cdc.gov/HRQOL/) reported 16 % for subjects with fair or poor self-rated health, with higher percentages for older subjects (>24 % among subjects aged over 65 years). For depressive symptoms, a recent study reported that roughly 11 % of the subjects in the primary care population had PHQ-2 scores 3 or higher [31]. We also found that the greatest contributor to HRQOL deficits was disease burden, especially for perceived general health (relative influence of 63 %). For depressive symptoms, disease burden and age had similar influence on the risk of deficits. The impact of disease burden on deficits of HRQOL likely reflects the impact of disease symptoms (like pain, shortness of breath) upon HRQOL. It may also reflect the relationship between disease burden and functional decline [32]. After accounting for disease burden and age, demographic characteristics showed minimal influence, regardless of individual strong association. While the importance of disease burden on HRQOL deficits has been supported in studies on patients with chronic diseases and/or more general population [21, 33–36], our study quantified the relative influence of disease burden when other commonly collected determinants such as demographic characteristics were simultaneously considered.

We observed that subjects with deficits in perceived general health were slightly older than those without deficits. However, age was inversely associated with risk of deficits in depressive symptoms. Major depressive symptoms have been reported as fairly uncommon in older adults [37, 38]. The incidence of depression tends to peak at age 30 and decrease thereafter with a small increase at age 50 [37, 38]. We also observed that lower level of education, ever smoking, and no alcohol consumption were associated with higher risk of HRQOL deficits. These findings are supported by others using more general populations [39–42]. Lower educational level can be a surrogate for lower socioeconomic status. The deficit in HRQOL may reflect the relationship between lower income and HRQOL. In the MCB, very few of our participants reported excessive drinking (86 % with one drink only per day), thus our population is primarily one of moderate drinkers vs. non-drinkers. Moderate drinking has been reported to have health benefits and may partially explain our finding [43, 44].

Employment status was observed to be associated with risk of HRQOL deficits, with different effect by gender. The association of currently not working for pay was stronger among males compared to females, even after adjusting for disease burden. This observation suggests that psychological stress related to unemployment may be higher for males than females and thus impact quality of life. It may also reflect the functional ability to continue to work with those with functional disabilities opting out of employment.

Obesity did impact deficit of HRQOL and did show some gender differences. For females, obesity is strongly associated with higher rate of HRQOL deficits, although there is no difference between normal and overweight females. Once adjusting for disease burden, obesity is no longer associated with HRQOL. Although overweight and obesity are socially undesirable, especially in females, its psychological impact may be minimal, considering roughly 70 % of the MCB participants were at least overweight. For males, overweight is negatively associated with the risk of deficits in HRQOL. The association remained after adjusting for disease burden. Such a finding is supported by several recent studies showed that being overweight is linked to better clinical outcomes, including survival [45–48].

There are some limitations to the study. First, there may be survival bias due to the use of prevalent diseases. Second, the MCB participants do not fully represent all patients seen at Mayo Clinic, as it does not include those who are healthiest (because they did not visit their primary care providers and thus were not invited) and the sickest (inability or refusal to participate). Third, comorbid health conditions are based on self-report, and thus there is potential for recall bias with self-report. Fourth, patients may have underlying health reasons of minimizing less desirable lifestyle attributes like alcohol or smoking. In addition, participants with no current alcohol consumption may include those who drank excessively before but now do not drink any longer. Lastly, a significant proportion (roughly 40 %) of the MCB participants is residents of Olmsted County, MN, where the Mayo Clinic Rochester is located. These residents are largely white and well educated which may limit some of the generalizability of the study to different populations.

## Conclusion

Comorbid health burden is the influential risk factor for deficits in perceived general health. For depressive symptoms, both age and comorbid health burden were equally important. Our findings suggest that healthcare providers may need to account for comorbid health burden and age for interpretation. HRQOL outcomes may need to be reported by different age category or by different comorbid health burden like Minnesota Medical Tier [25]. Categorizing both groups may allow accurate comparison of HRQOL which is not dependent on different comorbid health burden. As comorbid health burden is not easily changeable for the health system, healthcare providers should continue to emphasize the importance of prevention to potentially improve future HRQOL. The emphasis on lifestyle modification through weight management and exercise may be important to reduce the morbidity.

## Abbreviations

HRQOL: Health-related quality of life
QOL: Quality of life
MCB: Mayo Clinic Biobank
BMI: Body mass index
EMR: Electronic medical record
OR: Odds ratio
CI: Confidence interval
GBM: Gradient boosting machine
